# Contribution of reperfusion hemorrhage to T2 and T2* CMR in the quantification of hemorrhage extent and area-at-risk after acute myocardial infarction

**DOI:** 10.1186/1532-429X-15-S1-O104

**Published:** 2013-01-30

**Authors:** Nilesh R Ghugre, Mohammad I Zia, Idan Roifman, Bradley H Strauss, Kim A Connelly, Graham Wright

**Affiliations:** 1 Physical Sciences, Sunnybrook Research Institute, Toronto, ON, Canada; 2 Schulich Heart Research Program, Sunnybrook Health Sciences Centre, Toronto, ON, Canada; 3 Medical Biophysics, University of Toronto, Toronto, ON, Canada; 4 Cardiology, St. Michaels Hospital, Toronto, ON, Canada

## Background

In acute myocardial infarction (AMI), reperfusion injury is often associated with hemorrhage and microvascular obstruction (MVO), which are independent predictors of poor patient outcomes. T2 and T2* cardiovascular magnetic resonance (CMR) approaches have been instrumental in detecting hemorrhage; however, the relative sensitivity of each of these measures remains to be investigated. In this patient study, our objective was to quantitatively compare the extent of hemorrhage relative to the area-at-risk (AAR) post-AMI using T2 and T2* mapping techniques.

## Methods

50 patients with STEMI underwent a CMR exam on a 1.5T scanner (GE Signa Excite) at 48 hrs post percutaneous-coronary-intervention. T2 was quantified using a cardiac-gated T2-prepared spiral imaging sequence (TE=2.9-184 ms). T2* was determined from a multi-echo gradient-echo acquisition (TE=1.4-12.7 ms). Infarct/MVO was assessed using early gadolinium enhancement (EGE) imaging. Using T2* maps, hemorrhage extent was quantified as the region where T2* was at least two standard deviations (SD) below that in remote myocardium. From T2 maps, AAR was determined as the region where T2 was at least two SD above that in remote myocardium. Any ‘signal voids' within this area were identified as hemorrhage and the computed AAR was accordingly corrected. T2 and T2* values were recorded in these regions. Areas were expressed as a percentage of LV myocardium. Only patients with MVO were included in the analysis since hemorrhage is typically associated with an MVO.

## Results

**Figure 1:**
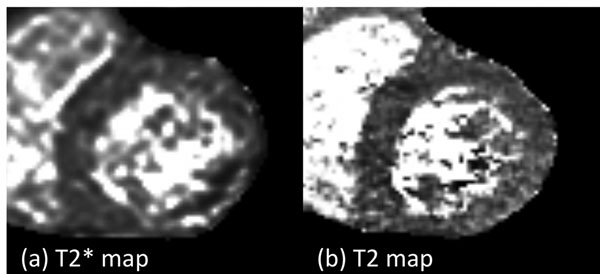
(a) Plot demonstrates relationship between percent hemorrhage as quantified from T2 and T2* maps and area-at-risk (AAR) computed from T2 maps. (b) Bar plot shows T2 and T2* relaxation times in various regions of the myocardium. Error bars represent standard deviation.

**Figure 2:**
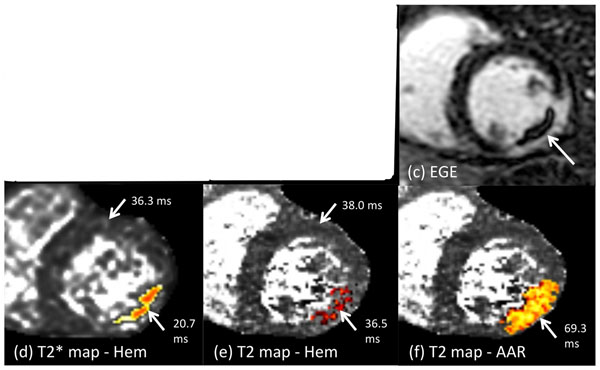
Representative short-axis images from a patient who underwent a CMR exam at 48 hrs post percutanious coronary intervention (PCI). Early gadolinium enhancement demonstrates a large transmural infarction with MVO in the left circumflex territory (c). The T2* map (a) quantified intramyocardial hemorrhage as hypointense regions (d) while the T2 map (b) identified hypointense regions (e) within the area-at-risk (AAR) shown in (f).

19 patients (38%) presented with an MVO. Hemorrhage extent determined by T2 was significantly less than that computed from T2* (4.1±2.8% vs. 10.0±7.5%, p=0.001). AAR assessed by T2 was underestimated if the hemorrhagic region was not accounted for (28% vs 32%, p<0.0001). Hemorrhage extent, by both T2 and T2*, demonstrated linear relationship with AAR (Fig. 1a), although slope of the T2*-AAR relationship was nearly three-fold greater than T2. In regions identified as hemorrhage, both T2 and T2* values were significantly lower than remote myocardium (Fig. 1b); however, the change was more dramatic in T2* (40%) than in T2 (4%). T2 was significantly elevated in the AAR (edema) and differences in T2 were also apparent in AAR and AAR excluding hemorrhagic regions (Fig. 1a). Fig. 2 shows an example from a representative patient.

## Conclusions

T2* appears to be a more sensitive and specific indicator of hemorrhage. T2 underestimates the hemorrhage extent and AAR due to counteracting influence from edema. In patients with MVO/hemorrhage, both T2 and T2* imaging may be necessary to accurately quantify hemorrhage extent and AAR.

## Funding

NA.

